# 16-channel SiPM high-frequency readout with time-over-threshold discrimination for ultrafast time-of-flight applications

**DOI:** 10.1186/s40658-023-00594-z

**Published:** 2023-12-04

**Authors:** Vanessa Nadig, Matthias Hornisch, Jakob Oehm, Katrin Herweg, Volkmar Schulz, Stefan Gundacker

**Affiliations:** 1 Department of Physics of Molecular Imaging Systems, Experimental Molecular Imaging, RWTH Aachen University, Forckenbeckstraße 55, 52074 Aachen, Germany; 2 Hyperion Hybrid Imaging Systems GmbH, Pauwelsstraße 19, 52074 Aachen, Germany; 3 III. Physikalisches Institut B, RWTH Aachen University, Otto-Blumenthal-Straße, 52074 Aachen, Germany; 4 Fraunhofer Institute for Digital Medicine MEVIS, Forckenbeckstraße 55, 52074 Aachen, Germany

**Keywords:** Time-of-flight, PET, Fast timing, High-frequency readout, TOFPET2 ASIC, CTR, TOT discrimination

## Abstract

**Background:**

Over the past five years, ultrafast high-frequency (HF) readout concepts have advanced the timing performance of silicon photomultipliers (SiPMs). The shown impact in time-of-flight (TOF) techniques can further push the limits in light detection and ranging (LiDAR), time-of-flight positron-emission tomography (TOF-PET), time-of-flight computed tomography (TOF-CT) or high-energy physics (HEP). However, upscaling these electronics to a system-applicable, multi-channel readout, has remained a challenging task, posed by the use of discrete components and a high power consumption. To this day, there are no means to exploit the high TOF resolution of these electronics on system scale or to measure the actual timing performance limits of a full detector block.

**Methods:**

In this work, we present a 16-channel HF readout board, including leading-edge discrimination and a linearized time-over-threshold (TOT) method, which is fully compatible with a high-precision time-to-digital converters (TDCs), such as the picoTDC developed at CERN. The discrete implementation allows ideal adaptation of this readout to a broad range of detection tasks. As a first step, the functionality of the circuit has been tested using the TOFPET2 ASIC as back-end electronics to emulate the TDC, also in view of its properties as a highly scalable data acquisition solution.

**Results:**

The produced board is able to mitigate influences of baseline shifts in the TOFPET2 front end, which has been shown in experiments with a pulsed laser, increasing the achievable intrinsic coincidence timing resolution (CTR) of the TOFPET2 readout electronics from 70 ps (FWHM) to 62 ps (FWHM). Single-channel coincidence experiments including a $\gamma$-source, 2$\times$2$\times$3 mm$^{3}$ LYSO:Ce,Ca crystals and Broadcom NUV-MT SiPMs resulted in a CTR of 118 ps (FWHM). For a 4$\times$4 matrix of 3.88$\times$3.88$\times$19 mm$^{3}$ LYSO:Ce,Ca crystals one-to-one coupled to a 4$\times$4 array of Broadcom NUV-MT SiPMs, an average CTR of 223 ps (FWHM) was obtained.

**Conclusion:**

The implemented 16-channel HF electronics are fully functionall and have a negligible influence on the timing performance of the back-end electronics used, here the TOFPET2 ASIC. The ongoing integration of the picoTDC with the 16-channel HF board is expected to further set the path toward sub-100 ps TOF-PET and sub-30ps TOF resolution for single-photon detection.

## Background

Ultrafast high-frequency readout electronics, when combined with fast scintillators and silicon photomultipliers (SiPMs), have been continuously pushing the state of the art in time-of-flight positron emission tomography (TOF-PET) toward sub-100 ps timing limits over the past five years [1–6]. Further fields of application concern light detection and ranging (LiDAR), time-of-flight computed tomography (TOF-CT) or high-energy physics (HEP). Current record coincidence time resolution (CTR) in TOF-PET detector configurations is observed with commercially available components such as 2$\times$2$\times$3 mm$^{3}$ Ce-doped and Ca-co-doped lutetium yttrium oxyorthosilicate (LYSO:Ce,Ca) crystals fabricated by Taiwan Applied Crystal (TAC) coupled to near-ultra-violet metal-in-trench (NUV-MT) SiPMs manufactured by Broadcom Inc., for which a CTR of 56 ps (FWHM) is reported [3]. Using 3$\times$3$\times$20 mm$^{3}$ LYSO:Ce,Ca crystals, this performance deteriorates to 95 ps (FWHM) due to the increased crystal length, but shows that the timing limits of scintillator and SiPM still permit a sub-100 ps CTR with potential components of clinical systems [3]. Yet, the HF readout electronics remain to be upscaled to truly transfer this performance to system level, leaving various challenges to tackle, such as power consumption and discrete components. Current TOF-PET systems achieve a CTR of few hundred picosecond on system level [7–12], where the Biograph Vision scanner by Siemens Healthineers realizes a CTR of 228 ps [13]. Latest announcements report a performance increase to 178 ps (FWHM) [14].

Until now, despite attempts to limit the power consumption of these high-bandwidth electronics [6, 15], a 16-channel version of HF readout electronics has not been implemented. Propositions to combine energy and timing channels [16] have shown to reduce the number of required readout channels, but also to increase the amount of components and circuit complexity. In addition, to ensure system applicability, adapting the circuit to the requirements of different, highly integrated back-end electronics, which employ time-to-digital converters (TDCs) and charge-to-digital converters (QDCs) for precise signal digitization, is the next imminent task.

Existing scalable readout electronics combining the analog front-end and digitization electronics in a highly integrated manner, such as the TOFPET2 ASIC by PETsys Electronics S.A. [17–24], have been used to digitize the signals of an HF readout circuit, which resulted in marginal improvement of the CTR [25]. Additionally, this approach revealed limitations of the TOFPET2 electronics in form of baseline shifts and side peaks in the coincidence time difference spectrum due to falsely assigned timestamps, which in turn causes a smeared TOF kernel, not suitable to digitize very fast or highly amplified signals [25].

This study shows a 16-channel HF readout implementation, including time-over-threshold (TOT) discrimination. We validate that the contribution of the newly designed front-end electronics does not influence the achievable timing limits of the scintillator and SiPM using digitization via an oscilloscope. In addition, we evaluate the design regarding its functionality in multi-channel TOF-PET detector readout combined with currently available back-end electronics such as the TOFPET2 ASIC. Coincidence experiments are performed with single and multiple channels using LYSO:Ce,Ca crystals and Broadcom NUV-MT SiPMs. The CTR is also evaluated using bismuth germanate (BGO) crystals with respect to exploiting the prompt emission of Cherenkov photons [26–30]. Furthermore, the incorporated discriminator is shown to mitigate the side peaks observed in the coincidence time difference spectra of the TOFPET2 ASIC. This first step in scaling HF electronics to 16 channels will give a handle to study the time resolution of different scintillator topologies and concepts, such as heterostructured and metascintillators [31–33], side-readout configurations [4], without a significant deterioration caused by the electronic readout. The designs are further adapted to be used with the recently developed picoTDC developed at CERN [34] with an intrinsic time resolution of below 10 ps.

**Fig. 1:**
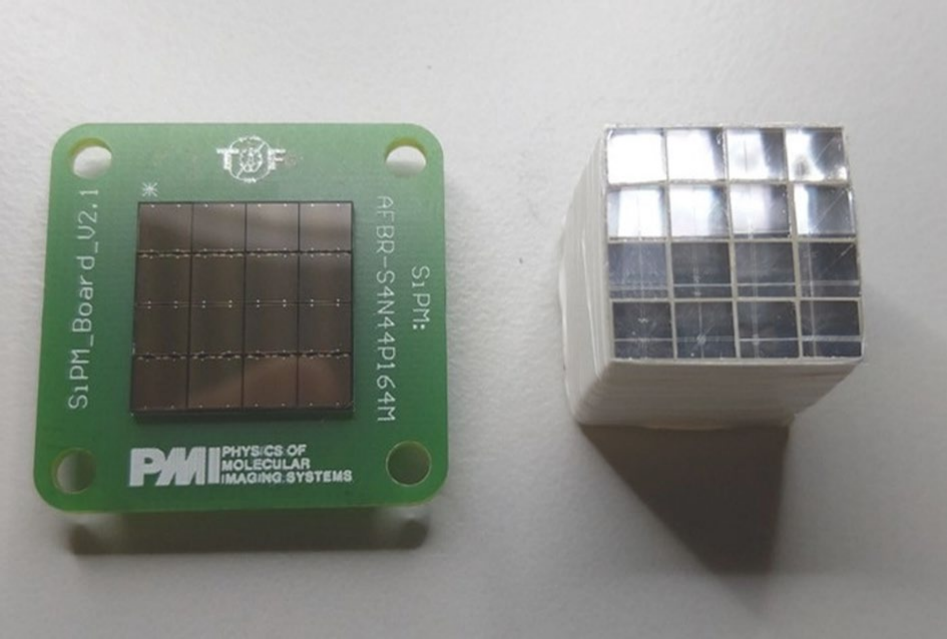
A detector block consisting of a $4\times 4$ Broadcom NUV-MT SiPM array, one-to-one coupled to a $4\times 4$ LYSO:Ce,Ca crystal matrix, where crystals were isolated by a 150-$\upmu$m layer of ESR-OCA-ESR sheets

## Materials

The following paragraphs provide details on materials and setups used to investigate the newly implemented HF readout. Apart from the scintillators and photosensors used (c.f. Sect. “Scintillators and photosensors”), these setups comprise:

1.: A single-channel version of the HF readout circuit developed in [1, 15] to provide performance benchmarks according to the state of the art (c.f. Sect. “Single-channel HF electronics without TOT discrimination featuring oscilloscope readout”). An oscilloscope is used for digitization.
2.: A single-channel version of the HF readout circuit developed in this study including a new discriminator (TLV3801 [35]) and digitization via an oscilloscope to evaluate the impact of the leading-edge discrimination on the performance (c.f. Sect. “Multi-channel HF readout electronics with TOT discrimination”).
3.: An identical multi-channel version of the circuit from 2., integrating 16 channels on one board and realizing a connection to the TOFPET2 ASIC as initial back end as well as the picoTDC demonstration board [34] (c.f. Sect. “Multi-channel HF readout electronics with TOT discrimination”).
4.: The TOFPET2 evaluation kit developed by PETsys Electronics S.A. [17] to be used as back end for the newly developed board from 3. and to provide performance benchmarks with a commercially available readout (c.f. Sect. “TOFPET2 ASIC evaluation kit”).
5.: A picosecond pulsed laser to illuminate the photosensors with optical photon pulses to study the intrinsic time resolution and multi-photon coincidence resolution of the TOFPET2 ASIC and the newly developed front end (c.f. Sect. “Optical irradiation with a picosecond pulsed laser”).

The ${}^{22}$Na source used in all $\gamma$-irradiation experiments had an activity of 1.8 MBq. The whole setup was placed in a dark chamber, which was regulated to 16 ${}^{\circ }\text {C}$ ambient temperature.

### Scintillators and photosensors

#### Reference detector and single-channel experiments

Single NUV-MT SiPMs (AFBR-S4N44P014M, 3.8$\times$3.8 mm$^{2}$, 40-mm SPAD pitch) were used in combination with single LYSO:Ce,Ca crystals of 2$\times$2$\times$3 mm$^{3}$ and 3$\times$3$\times$19 mm$^{3}$ size, manufactured by TAC. Additional coincidence experiments were performed using the same NUV-MT SiPMs (3.8$\times$3.8 mm$^{2}$, 40-mm SPAD pitch) coupled to single BGO crystals of 2$\times$2$\times$3 mm$^{3}$ size, manufactured by EPIC crystal.

One of the two single Broadcom NUV-MT SiPMs coupled to a 2$\times$2$\times$3 mm$^{3}$ LYSO:Ce,Ca crystal acted as reference detector in all further multi-channel coincidence experiments. All scintillators and SiPMs have been optically coupled using Cargille Meltmount^™^ (*n* = 1.582) and were wrapped in Teflon tape to ensure a high light output [36].

In prior studies to investigate the intrinsic timing resolution of the TOFPET2 ASIC (CTR$_\textsf{int}$), two LYSO:Ce crystals (EPIC crystal, 2$\times$2$\times$3 mm$^{3}$) mounted on Broadcom AFBR-S4N33C013 SiPMs (3$\times$3 mm$^{2}$, 30-$\upmu$m SPAD pitch) were employed [25]. These studies have been repeated and extended in this work using Broadcom NUV-MT SiPMs for consistency.

#### A one-to-one coupled LYSO:Ce,Ca detector block

In multi-channel experiments involving 16 channels, a Broadcom NUV-MT SiPM array (AFBR-S4N44P164M, 3.8$\times$3.8 mm$^{2}$, 4$\times$4 channels, 40-$\upmu$m SPAD pitch) was employed in combination with a self-assembled 4$\times$4 array of 3.8$\times$3.8$\times$19-mm$^{3}$ LYSO:Ce,Ca crystals optically separated by a stack of enhanced specular reflector (ESR) and optical clear adhesive (OCA) foils fabricated by 3 M^™^ (c.f. Fig. 1). The thickness of the entire stack of ESR-OCA-ESR sheets was 155 $\upmu$m. The same sheets were used to cover the outer walls of the detector block. For protective reasons, the assembled crystal matrix was wrapped into several layers of Teflon tape. Again, Cargille Meltmount^™^ (*n* = 1.582) was used to achieve optical coupling between SiPMs and scintillators. It has to be noted that in one direction of the detector block, this results in slight shift from one-to-one coupling due to the trenches for the bond wires of the SiPM array.

**Fig. 2:**
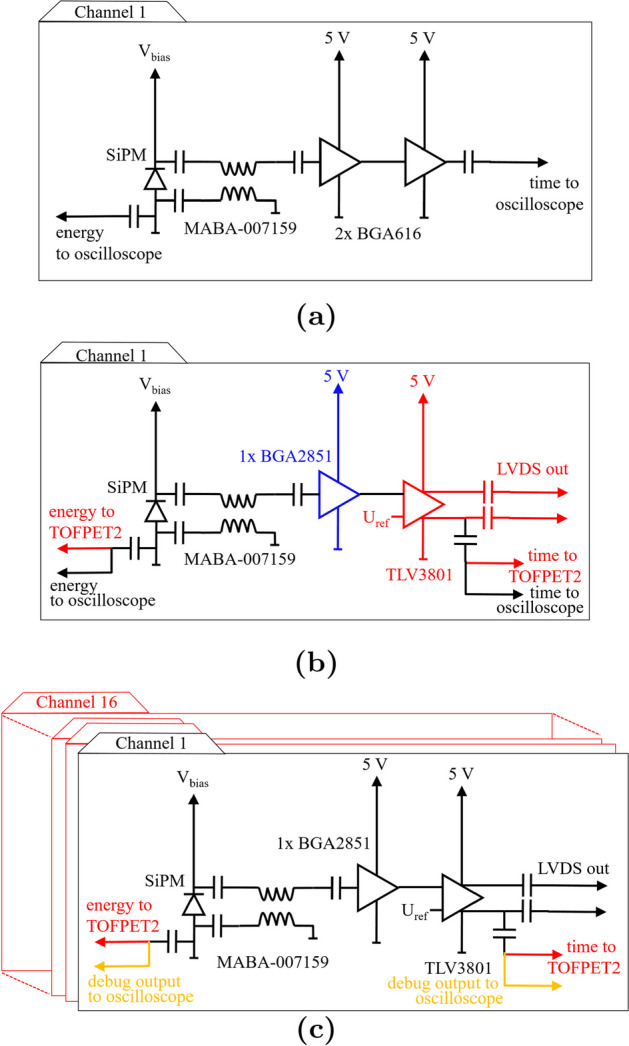
This figure illustrates the transition between setups and circuits used in this study. **a** Single-channel HF readout as developed by [1] and modified by [15]. **b** Single-channel HF readout including a TLV3801 for pulse discrimination. **c** Schematic circuit of a single channel of the 16-channel HF readout including the TLV3801 and establishing a connection to the TOFPET2 ASIC

**Fig. 3:**
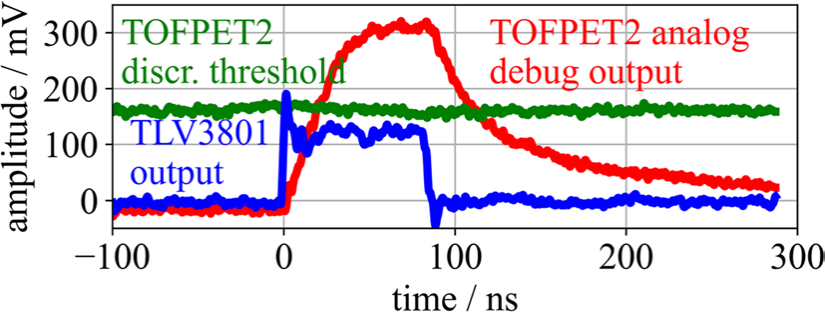
Signal waveforms at the output of the TLV3801 (blue) and the analog debug output of the TOFPET2 ASIC (red). The applied TOFPET2 discriminator threshold is drawn in green. These waveforms were acquired with a reduced transimpedance amplifier gain $\mathsf {G_T}$=375 $\Omega$ for the FEB/A of the TOFPET2 ASIC evaluation kit

**Fig. 4:**
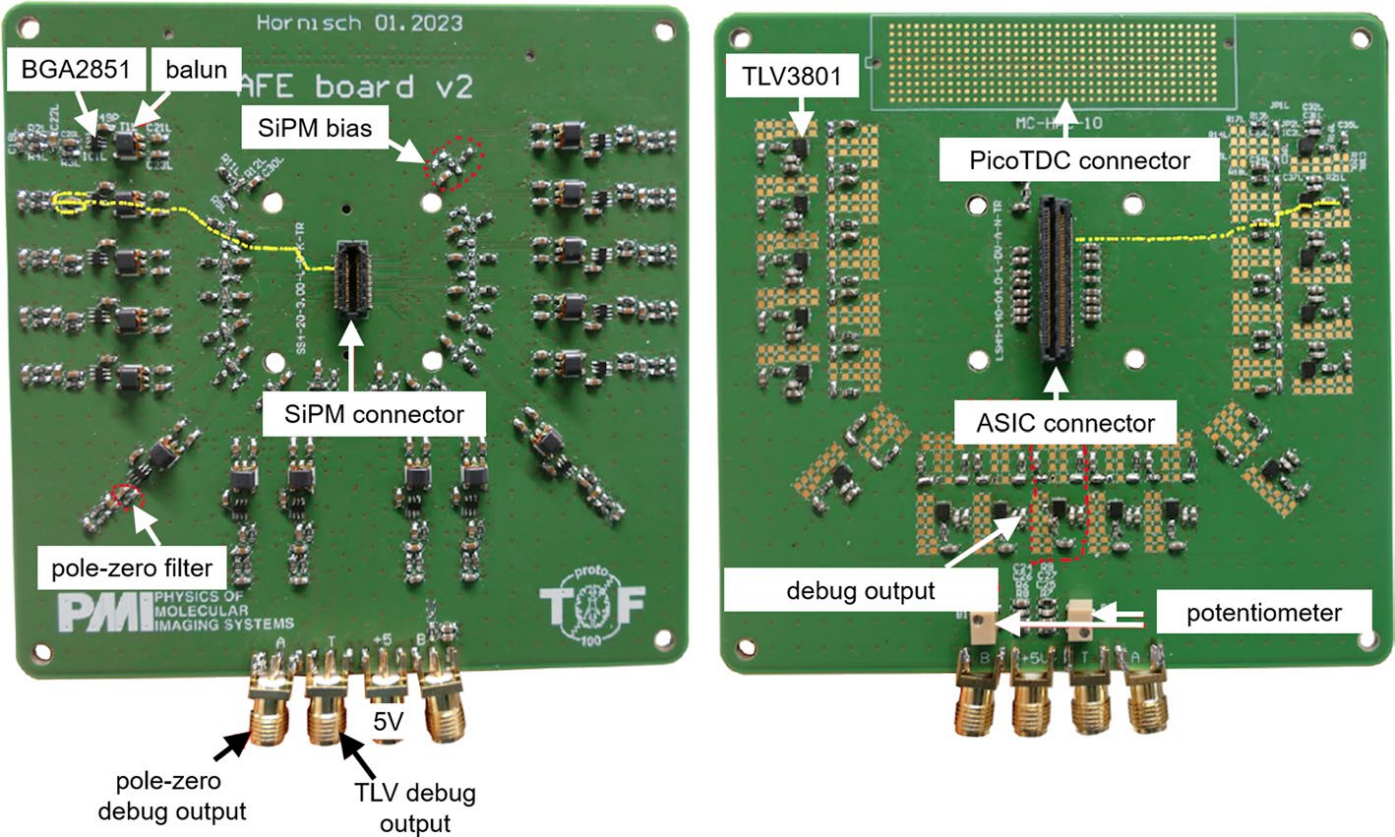
Manufactured PCB of the 16-channel HF readout including TOT discrimination. The track of one signal is shown in yellow

### Single-channel HF electronics without TOT discrimination featuring oscilloscope readout

For comparison to state-of-the-art single-channel readout, an HF circuit with the SiPM signal split to one timing and one energy channel per SiPM (c.f. Fig. 2a) was used in this study. The timing channel features a 3-GHz balun transformer (MABA-007159, turn ratio 1:1, Macom [37]) and two BGA2851 (NXP Semiconductors [38]) amplifiers and the energy channel employs an AD8000 operational amplifier. The timing signal was shaped with capacitive filters to minimize baseline shifts. An oscilloscope (Lecroy Waverunner 9404 M-MS [39], bandwidth 4 GHz, 20 GS s$^{-1}$) is used to read out the timing and energy channels, triggering on coincidences. Bias and threshold scans were performed with all detector configurations.

### Single-channel HF electronics with TOT discrimination featuring oscilloscope readout

To evaluate the impact of additional leading-edge-discrimination of the timing signal on the achievable timing performance, a single-channel version of the HF electronics described in Sect. “Single-channel HF electronics without TOT discrimination featuring oscilloscope readout” was implemented featuring a TLV3801 comparator (Texas Instruments [35]) to perform leading-edge discrimination on the analog timing signal with an adjustable threshold (c.f. Fig. 2b). The TLV3801 is a high-speed comparator with a bandwidth of 3 GHz and a low-voltage differential signaling (LVDS) output [35], which was measured to allow for a leading-edge discrimination of 10 ps. An analysis of the signal characteristics of the HF readout electronics without TOT discrimination is provided in [15]. The number of BGA2851 amplifiers in the timing channel was reduced to one, which is followed by a pole-zero filter to reduce baseline shifts [40]. TLV3801 and BGA2851 are supplied with a voltage of 5 V, where all 16 channels together draw a current of 910 mA, resulting in a power consumption of 284 mW per channel due to the HF circuit, of which the major part is consumed by the TLVs. The TLV output, as depicted in Fig. 3, is routed to a Samtec LSHM connector for timestamp digitization via the TOFPET2 ASIC with leading-edge discrimination. An oscilloscope (Lecroy Waverunner 9404 M-MS [39], bandwidth 4 GHz, 20 GS s$^{-1}$) is again used to read out the timing and energy channels, triggering on coincidences. Additionally, this board allows to connect the outputs of the timing and energy channels to the TOFPET2 ASIC. Bias and threshold scans were performed with all detector configurations and both oscilloscope and TOFPET2 readout.

### Multi-channel HF readout electronics with TOT discrimination

We fabricated a printed circuit board (PCB) implementing a 16-channel HF readout with each channel equivalent to the circuit described in Sect. “Single-channel HF electronics with TOT discrimination featuring oscilloscope readout”. (c.f. Fig. 2c and Fig. 4). The outputs of all channels are routed into an LSHM connector, enabling the readout by the TOFPET2 ASIC. Additionally, the differential LVDS output of the TLV3801 is fed to a MC-HCP-10 connector, preparing the readout with the novel picoTDC developed at CERN [34]. Exchanging the connection interface, the use of other high-precision back-end electronics is possible. As the picoTDC does not feature a charge integration mode, a circuit design for a TOT energy measurement was implemented with and without linearization and will be tested using energy digitization via the TOFPET2 ASIC.

### TOFPET2 ASIC evaluation kit

For reference measurements with direct coupling (DC) in single- and multi-channel coincidence experiments, the TOFPET2 ASIC evaluation kit by PETsys Electronics S.A. (TOFPET2 ASIC version 2c [41–43]) was used in its standard configuration, employing a custom sensor front-end board (FEB/S), which enables a direct connection of the SiPM to the TOFPET2 ASIC input stage. Additional components of the evaluation kit used in this study are two interface front-end boards (FEB/I) connecting the ASIC to the development front-end board (FEB/D_v2) via flexible HQCD cables, a HV-DAC mezzanine board generating the SiPM bias voltage and a Gigabit Ethernet (GbE) board routing data to the laboratory computer. The evaluation kit is also used as initial back end for the newly implemented HF circuit (c.f. Sects. “Single-channel HF electronics with TOT discrimination featuring oscilloscope readout” and “Multi-channel HF readout electronics with TOT discrimination”).

### Optical irradiation with a picosecond pulsed laser

**Fig. 5:**
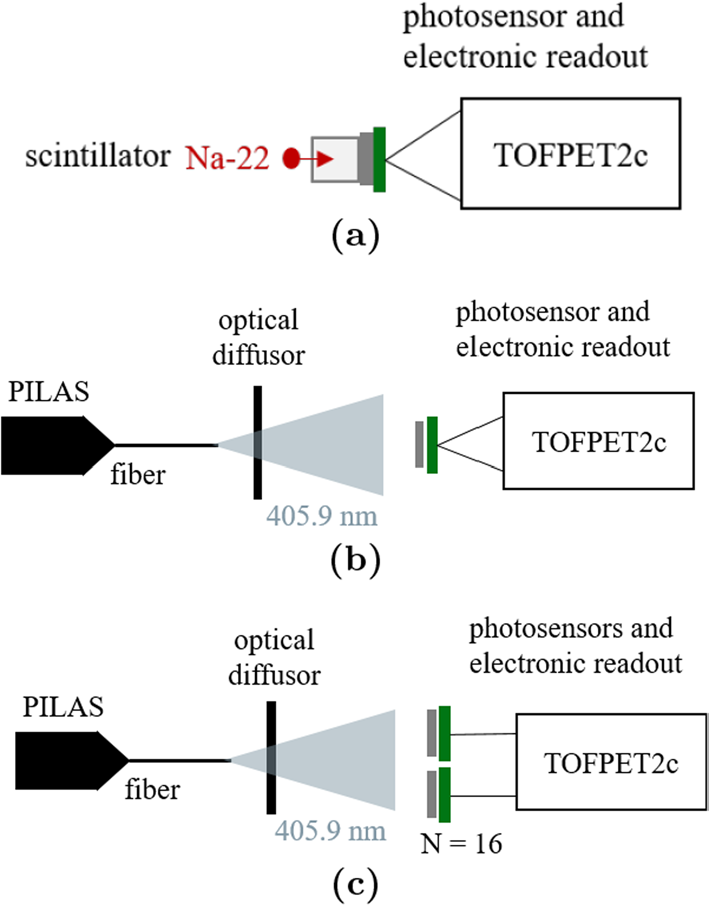
Different SiPM and ASIC channel combinations realized to investigate the contribution of the electronic front end in measurements with SiPM irradiation via scintillation emission and optical photons generated by a picosecond pulsed laser (PILAS). **a** and **b** One SiPM channel coupled to two ASIC channels of the same ASIC. **c** Two out of 16 SiPM channels each coupled to one ASIC channel of the same ASIC and optically illuminated

Coincidence experiments with $\gamma$-irradiation were complemented by illuminating the bare SiPMs with optical photon pulses of 406 nm wavelength using a picosecond pulsed laser (PILAS) from NKT Photonics instead of relying on $\gamma$-excitation of a scintillator. The advantage lays in a short, well-defined pulse of optical photons, depositing the same charge at a desired event rate. The setup consists of a laser head and fiber and an optical diffusor (c.f. Fig. 5). Pulse frequencies between 0.1 kHz and 50 kHz were used to resemble the event rate in a coincidence experiment with a $\gamma$-source.

**Fig. 6:**
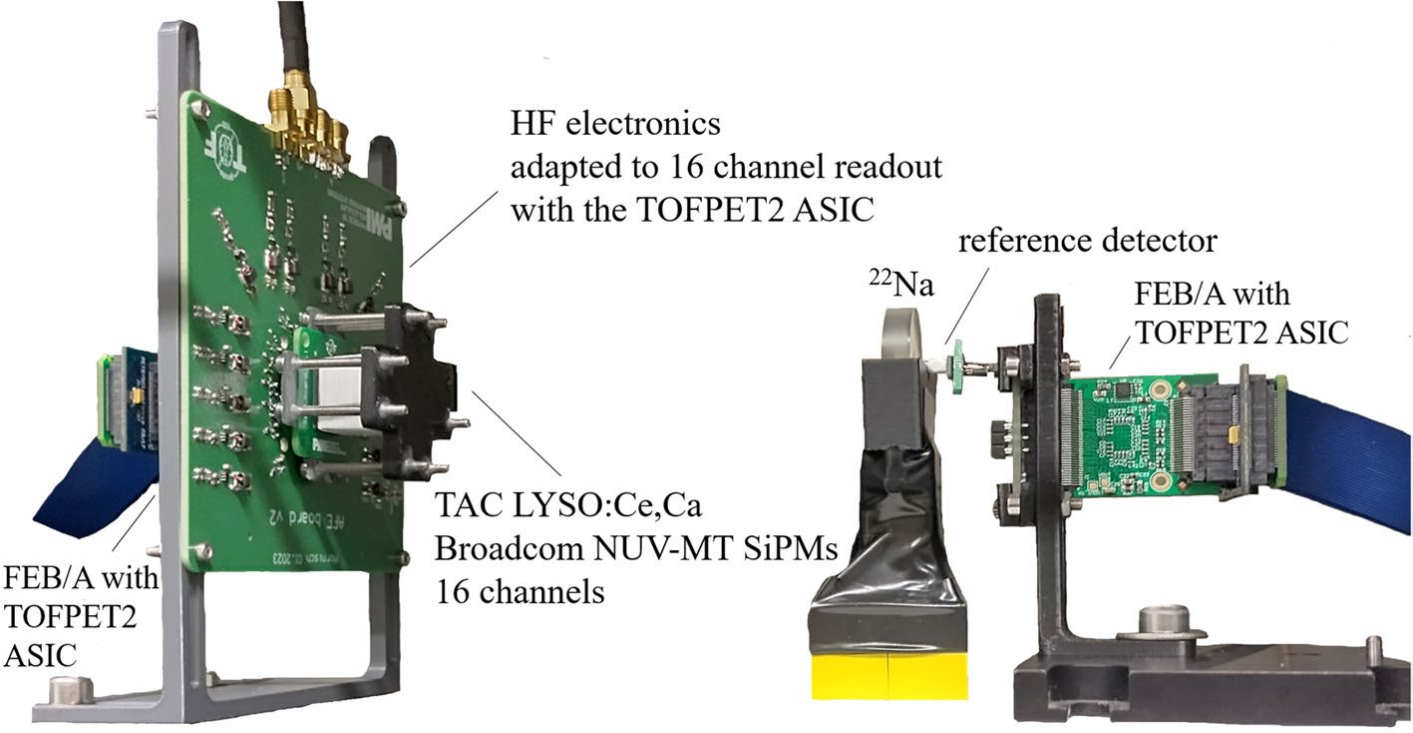
16-channel HF readout board including TOT discrimination and set up in a coincidence experiment with a reference detector using a ${}^{22}$Na source

**Fig. 7:**
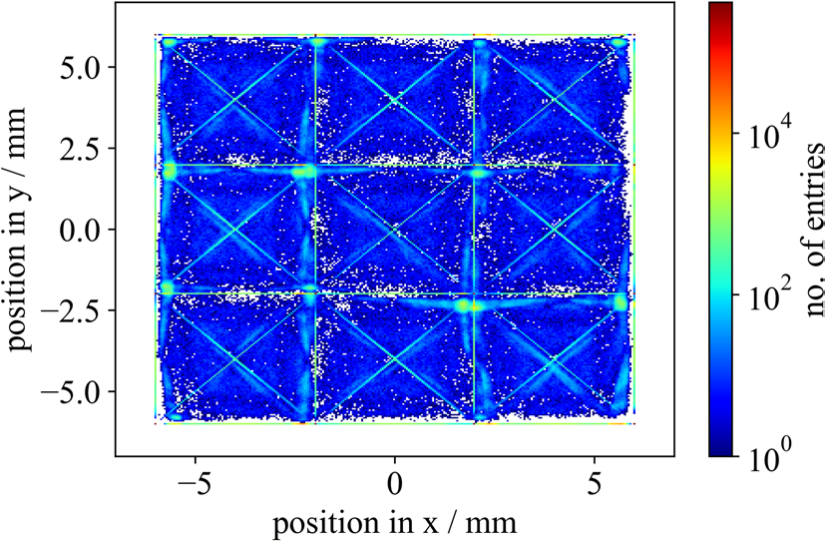
Computed COG of a detector block consisting of a $4\times 4$ Broadcom NUV-MT SiPM array one-to-one coupled to a $4\times 4$ LYSO:Ce,Ca crystal matrix at a bias voltage of 41.5 V (overvoltage 9 V) and a discriminator threshold of $\mathsf {vth\_t1}$=50. The COG was computed only considering the neighboring channels of the hottest pixel. Gaps in horizontal orientation correspond to the trenches for the bond wires of the NUV-MT SiPM array [44], opposed to an exactly symmetric 4$\times$4 crystal matrix

## Methods

The following paragraphs provide details on the experimental setups, data acquisition and processing methods applied in this study. A single Broadcom NUV-MT SiPM coupled to a 2$\times$2$\times$3-mm$^{3}$ LYSO:Ce,Ca crystal acted as reference detector in all single- and multi-channel coincidence experiments. The conducted experiments comprise:

1.: Single-channel coincidence experiments using a single-channel version of the state-of-the-art HF readout circuit without TOT discrimination read out via an oscilloscope. This has the objective to provide a benchmark on the achievable CTR in accordance with prior studies [3] (c.f. Sect. “Performance benchmarks”).Additional performance benchmarks were acquired establishing a direct connection of the SiPM signals to the TOFPET2 ASIC.
2.: Single-channel coincidence experiments using a single-channel version of the HF readout circuit including TOT discrimination and TOFPET2 readout. This has the objective to characterize the impact of the TOT discrimination on the achievable CTR with a commercial back end in comparison with prior studies [25] (c.f. Sect. “Single-channel experiments to characterize the impact of the TOT discrimination on the CTR”).
3.: Single-channel experiments using the 16-channel version of the HF readout circuit including TOT discrimination and oscilloscope readout. This has the purpose to study the effect of increased electronic crosstalk and validate the performance of the newly designed board (c.f. Sect. “Single-channel experiments to characterize the impact of the scaling on the CTR”).
4.: Multi-channel experiments using the 16-channel version of the HF readout circuit including TOT discrimination and oscilloscope readout. This has the purpose to study the effect of increased crosstalk and light-sharing on the performance of the HF circuit (c.f. Sect. “Single-channel experiments to characterize the impact of the scaling on the CTR”).
5.: Multi-channel experiments using the 16-channel version of the HF readout circuit including TOT discrimination and TOFPET2 readout. This has the purpose to evaluate the multi-channel performance of the HF circuit (c.f. Sect. “Multi-channel coincidence experiments with head-on irradiation using 16-channel HF electronics and digitization via the TOFPET2 ASIC”).
6.: Experiments with a pulsed laser with the objective to determine the intrinsic timing resolution and multi-photon coincidence time resolution of the TOFPET2 ASIC (c.f. Sect. “Laser irradiation to investigate the timing limits of the 16-channel HF electronics connected the TOFPET2 ASIC”).

### Performance benchmarks

We used the state-of-the-art HF readout and conducted bias and threshold scans for a range of thresholds between 10 mV and 300 mV for the single-channel detector configurations to establish performance benchmarks in terms of CTR. The bias voltages for the SiPMs were provided by a Keithley 2400 Sourcemeter [45]. The coincidences were digitized via a Lecroy Waverunner 9404 M-MS oscilloscope (bandwidth 4 GHz, 20 GS s$^{-1}$) [39]. The same experiments were conducted employing a system-applicable, multi-channel readout, namely the TOFPET2 ASIC designed by PETsys Electronics S.A., which was used together with its evaluation kit. After calibrating the channel baselines, TDCs and charge-to-digital converters (QDCs) using the calibration routine provided with the evaluation kit software, bias and threshold scans were performed with a reduced input stage impedance of approximately 11 $\Omega$ ($\mathsf {fe\_ib1}$ = 0) and with default trigger configuration (three-threshold trigger logic). The second timing threshold $\mathsf {vth\_t2} = 20$ and the energy threshold $\mathsf {vth\_e} = 15$ are kept constant, while the first timing threshold $\mathsf {vth\_t1}$ is varied using a least significant bit (LSB) value of 6.66 mV. In all experiments, the ASIC is operated in charge-integration mode (QDC mode), determining the signal energy via signal integration over a period of 290 ns. Using the $\mathsf {convert\_raw\_to\_singles\_method}$ implemented by PETsys Electronics S.A., acquired raw data are converted to single-hit information, each with a timestamp, energy value and channel ID. For the TOFPET2 ASIC, we as well acquired data with the self-assembled detector block to provide a multi-channel CTR benchmark in addition to the single-channel performance.

### Single-channel experiments to characterize the impact of the TOT discrimination on the CTR

The impact of adding TOT discrimination to the HF readout concept underwent performance tests in a single-channel readout configuration in a single-channel board version. In these experiments, the threshold of the TLV3801 was varied, whereby a single-SPAD amplitude level corresponds to about 10 mV at an overvoltage of 9 V using Broadcom NUV-MT SiPMs. Using the original HF readout circuit without the TLV, we usually trigger on a range of thresholds between 10 mV and 300 mV. With LYSO:Ce,Ca and Broadcom NUV-MT SiPMs, optimal performance is usually observed at thresholds between 40 mV and 90 mV. Taking into account the reduced amplification factor by omitting one BGA2851 amplifier, the TLV trigger threshold is still in a range where we would expect optimal performance with the oscilloscope. Bias scans were performed using the HF readout including TOT discrimination and digitization via a Lecroy Waverunner 9404 M-MS oscilloscope (bandwidth 4 GHz, 20 GS s$^{-1}$) [39]. A threshold of 70 mV was used to trigger on the discriminated signal after the TLV3801. Results were compared to the state-of-the-art HF readout by [1, 15] and the direct readout with the TOFPET2 ASIC (c.f. “Performance benchmarks” section).

### Single-channel experiments to characterize the impact of the scaling on the CTR

To investigate the impact of the scaling of the circuitry on the CTR, additional measurements have been taken using the 4$\times$4 NUV-MT SiPM array, biasing all 16 SiPMs, but only coupling one 2$\times$2$\times$3 mm$^{3}$ or one 3.88$\times$3.88$\times$19 mm$^{3}$ LYSO:Ce,Ca crystal to one of the SiPMs, ruling out a deterioration solely due to electronic crosstalk between channels or the implementation of the 16-channel HF circuit. The experiments were conducted with the TOFPET2 ASIC as well as the oscilloscope as back end. Additionally, the CTR of a single channel of the 16-channel, one-to-one coupled detector block was measured with HF readout and digitization via the oscilloscope.

### Multi-channel coincidence experiments with head-on irradiation using 16-channel HF electronics and digitization via the TOFPET2 ASIC

In multi-channel experiments (c.f. Fig. 6), i.e., for in total 16 channels of the HF readout approach, the TOFPET2 ASIC designed by PETsys Electronics S.A. has been employed as back end, which was used together with its evaluation kit. Bias and TLV as well as ASIC threshold scans were conducted. The evaluation kit was operated using the same configuration and steps as described above. To reduce the impact of noise on the TOT discrimination, the transimpedance amplifier gain in the timing branch of the TOFPET2 ASIC was reduced to its minimum ($\mathsf {postamp\_gain\_t}$ = 375 $\Omega$) for all timing channels, but not the energy channels, for which the data acquisition routine $\mathsf {acquire\_sipm\_data}$ was altered. The circuit was again operated with an input stage impedance of $R_\textsf{in}=$11 $\Omega$ (parameter $\mathsf {fe\_ib1}$). Results were compared to a 16-channel DC readout of the same detector block with the TOFPET2 ASIC (c.f. Sect. “Performance benchmarks”).

### Laser illumination to investigate the timing limits of the 16-channel HF electronics connected the TOFPET2 ASIC

Single NUV-MT SiPMs, connected to the TOFPET2 ASIC, were optically irradiated with the TOFPET2 input stage impedance configured to $R_\textsf{in}=$11 $\Omega$ (parameter $\mathsf {fe\_ib1}$). For all measurements, the same NUV-MT SiPM array was used. Successively, one SiPM was connected to two ASIC channels of the same ASIC (c.f. Figs. 5a and 5b, also demonstrated in [25]) and two SiPMs were each connected to one channel of the same ASICs (c.f. Fig.  5c). If illuminated with a laser, or the first case the intrinsic multi-photon coincidence time resolution (MPCTR$_\textsf{intr}$) is measured, for the latter case the time resolution will be referred to as multi-photon coincidence time resolution (MPCTR). Afterward, a 4$\times$4 Broadcom NUV-MT array connected to the multi-channel HF readout electronics was illuminated in the same way to evaluate the impact the changed front end has on the overall CTR. Pulse frequencies were varied in a range of 0.1 kHz to 100 kHz (equidistant triggers) to mimic the coincidence rate in $\gamma$-irradiation experiments.

### Data processing

For a detector block with 16 channels, the obtained data were clustered with a time window of 15 ns, which refers to performing an event search and identifying multiple hits that belong to the same $\gamma$-interaction. A center of gravity (COG) flood map was computed by weighting the position of the firing SiPMs with the energy digitized in the respective energy channel. An example of such a flood map is shown in Fig. 7. Clusters were assigned to a crystal by identifying the main pixel, i.e., the channel in the matrix with the highest energy signal. This step is omitted for all single-channel measurements.

Coincidences with the reference detector were matched within a coincidence time window of 7.5 ns considering each pair of timing and energy channel individually. The coincident $\gamma$-events were selected within $\pm 2\sigma$ around the photopeak in the raw energy value spectra of the main pixel. Coincidence time differences are computed from the timestamps in the timing channel in the case of all HF experiments and from the solely available channel per SiPM in standard TOFPET2 readout (DC readout). The CTR is determined as the FWHM of a Gaussian fitted to the coincidence time difference spectrum of the filtered $\gamma$-events. As this is done individually for each main pixel, a timeskew correction is not necessary. All values stated are corrected for the reference CTR. In case of coincidence experiments with BGO crystals, the shape of the coincidence time difference spectrum resembles two Gaussians, which are fitted as a function *G*(*x*) by weighting two normalized Gaussian $g_i(x)$ defined via [26, 27]

1 $$\begin{aligned} g_i(x) = \frac{1}{\sqrt{2\pi }\cdot \sigma _i}\cdot \exp {\left( -\frac{(x-\mu _i)^2}{2\sigma _i^2}\right) } \end{aligned}$$

using

2 $$\begin{aligned} G (x) = F_1 \cdot g_1 (x) + F_2 \cdot g_2 (x) \end{aligned}$$

with the weights normalized according to

3 $$\begin{aligned} \sum _{i=1}^2 F_i = 1 \end{aligned}$$

The resulting CTR is stated as the numerically calculated FWHM of the two summed Gaussians, whereby it is possible to specify the contribution of a faster and a slower component by the weights $F_i$.

The energy spectra were calibrated using the positions of the peaks at 511 keV and 1275 keV in the ^22^Na spectrum and a saturation correction model [21]

4 $$\begin{aligned} E = c \cdot s \cdot \log \left( \frac{1}{1-\frac{e}{s}} \right) , \end{aligned}$$

where *e* is the raw energy value in arbitrary units, *E* is the saturation-corrected energy value, *c* is a calibration factor, and *s* is the maximum saturated energy *e* possible. Afterward, the energy resolution was determined as the FWHM of the photopeak in the calibrated energy spectrum.

## Results

The following paragraphs report results on the coincidence experiments conducted with the different setup configurations introduced above. Table 1 provides an overview over all results obtained, listing the CTRs achieved in:

- Reference measurements with single LYSO:Ce,Ca crystals (2$\times$2$\times$3 mm$^{3}$) on Broadcom NUV-MT SiPMs connected to single-channel HF readout without TOT discrimination and with digitization via an oscilloscope (c.f. Sect. “Reference benchmarks in terms of CTR in γ-excitation experiments”)
- Reference measurements with the same detectors connected directly to the TOFPET2 ASIC (c.f. Sect. “Reference benchmarks in terms of CTR in γ-excitation experiments”)
- Performance measurements with the same detectors connected to single-channel HF readout with TOT discrimination and with digitization via an oscilloscope or the TOFPET2 ASIC (c.f. Sect. “Impact of the TOT discrimination on the HF performance in γ-radiation experiments”)
- Reference and performance measurements using the same photosensors and readout electronics as mentioned above together with two single BGO crystals with a size of 2$\times$2$\times$3 mm$^{3}$ (c.f. Sect. “Impact of the TOT discrimination on the HF performance in γ-radiation experiments”)
- Performance measurements with a single
  LYSO:Ce,Ca crystal of different size coupled to one channel of a 4$\times$4 SiPM matrix read out by multi-channel HF readout with TLV and with digitization via an oscilloscope (c.f. Sect. “Investigation on the performance of single channels in 16-channel HF electronics”)
- Performance measurements with the same detectors connected directly to the TOFPET2 ASIC (c.f. Sect. “Investigation on the performance of single channels in 16-channel HF electronics”)
- Performance measurements with the same detectors read out by multi-channel HF readout with TLV and with digitization via an oscilloscope or the TOFPET2 ASIC (c.f. Sect. “Investigation on the performance of single channels in 16-channel HF electronics”)
- Performance measurements with a 4$\times$4 LYSO:Ce,Ca crystal matrix connected to a 4$\times$4 SiPM matrix connected directly to the TOFPET2 ASIC (c.f. Sect. “16-channel HF electronics with a one-to-one coupled LYSO:Ce,Ca detector block”)
- Performance measurements with the same detectors read out by multi-channel HF readout with TLV and with digitization via an oscilloscope or the TOFPET2 ASIC (c.f. Sect. “16-channel HF electronics with a one-to-one coupled LYSO:Ce,Ca detector block”)

### Reference benchmarks in terms of CTR in $\gamma$-excitation experiments

With 2$\times$2$\times$3 mm$^{3}$ LYSO:Ce,Ca crystals and Broadcom NUV-MT SiPMs, the state-of-the-art version of the HF electronics read out by an oscilloscope shows CTRs of about 60 ps (FWHM) if tested without TOT discrimination. Using the TOFPET2c ASIC as an initial candidate as back-end electronics, a CTR of 120 ps was established as benchmark for the same crystals and NUV-MT SiPMs with standard TOFPET2 readout, excluding further signal amplification or discrimination prior to its input. The performance benchmarks can be found in Table 1 as well.

**Fig. 8:**
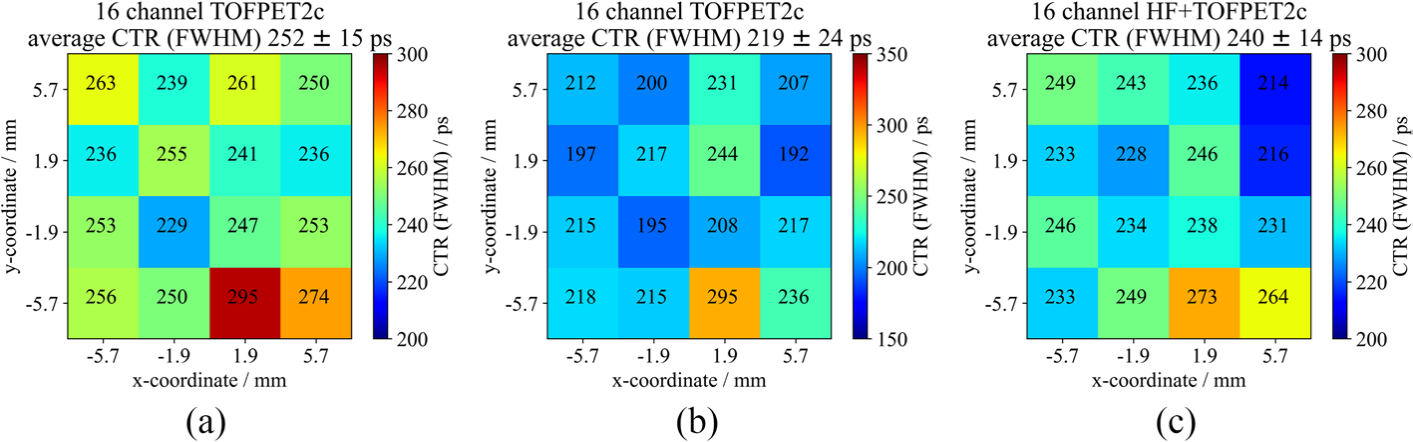
CTR of individual channels of a detector block consisting of a $4\times 4$ Broadcom NUV-MT SiPM arrays one-to-one coupled to a $4\times 4$ LYSO:Ce,Ca crystal matrix at a bias voltage of 41.5 V and a discriminator threshold of $\mathsf {vth\_t1}$=  50. **a** DC readout at default TOFPET2 configuration with R$_\textsf{in}$ = 30 $\Omega$. **b** DC readout with R$_\textsf{in}$ = 11 $\Omega$. **c** Upscaled HF readout including TOT discrimination via a TLV3801 with a threshold of 90 mV

### Impact of the TOT discrimination on the HF performance in $\gamma$-radiation experiments

With 2$\times$2$\times$3 mm$^{3}$ LYSO:Ce,Ca crystals and Broadcom NUV-MT SiPMs, compared to the reference measurement with CTRs of about 60 ps (FWHM) if tested without TOT discrimination (state-of-the-art HF readout), the initial single-channel board version read out by an oscilloscope shows CTRs of about 70 ps (FWHM) with the TLV3801 at low TLV thresholds (HF readout including TOT discrimination). Increasing the TLV threshold deteriorates the performance to 90±1 ps (FWHM) for a very high TLV threshold of 200 mV (c.f. Table 1), which corresponds to triggering on an amplitude of about 20 times the single-SPAD signal at an overvoltage of 9 V.

The HF circuit including TOT discrimination could not be operated at very low TLV thresholds lower than 40 mV in combination with the TOFPET2 ASIC due to instabilities (oscillations and feedback loops) as well as baseline shifts, which will be discussed in detail in Sect. "Single‑channel HF electronics without TOT discrimination featuring oscilloscope readout". For a threshold of 40 mV, the performance deteriorated from 70 ps (FWHM) with signal digitization via an oscilloscope to 118 ps (FWHM) with TOFPET2 readout. For a threshold of 90 mV, the performance deteriorated from 78 ps (FWHM) to 121 ps (FWHM), comparing the same types of measurements.

Comparing to the reference measurement without the HF readout circuit and TOT discrimination in front of the ASIC input stage, the same performance of about 120 ps (FWHM) is achieved.

Using crystals of clinical length (3$\times$3$\times$19 mm$^{3}$), a CTR of 161 ps (FWHM) could be achieved with TOFPET2 readout and the HF circuit with a TLV threshold of 90 mV (c.f. Table 1), which resembles the performance of 157±1 ps (FWHM) achieved with standard TOFPET2 readout in prior studies [3].

Using 2$\times$2$\times$3 mm$^{3}$ BGO crystals on 3.8$\times$3.8 mm$^{2}$ NUV-MT SiPMs and digitizing the discriminated signals of the HF circuit via the TOFPET2 ASIC, the CTR was measured to 542 ps (FWHM), which is worse than a CTR of 480 ps (FWHM) achieved for DC coupling (c.f. Table 1). In comparison, a CTR of 359 ps is measured if the signals of the 16-channel HF readout are digitized via an oscilloscope (c.f. Table 1), which is worse than the results achieved for BGO and HF readout in prior studies [29]. This deterioration can be attributed to the considerably high TLV threshold applied (ranging from 40 mV to 90 mV) in comparison to a single-SPAD amplitude of about 10 mV.

**Table 1:** CTR achieved with Broadcom NUV-MT SiPMs and the HF readout circuit and different back-end electronics at optimum bias settings and trigger threshold settings

Standard HF or TOFPET2 readout				
Crystal material and size	No. of channels	CTR$_\textsf{osci}$ (FWHM) / ps	CTR $_\textsf{TOFPET2}$ (FWHM) / ps	
TAC LYSO:Ce,Ca				
2$\times$2$\times$3 mm$^{3}$	1	58 ± 1	128 ± 2	
3$\times$3$\times$19 mm$^{3}$	1	95 ± 2 [3]	157 ± 1 [3]	
2$\times$2$\times$3 mm$^{3}$	1 of 16^‡^	n.a.	120 ± 1	
3.88$\times$3.88$\times$19 mm$^{3}$	16	n.a.	219 ± 24	
EPIC BGO				
2$\times$2$\times$3 mm$^{3}$	1	117$^{\circ }$	480	
		101 (57 %) / 285 (43 %)	342 (30 %) / 921 (70 %)	

16-channel HF readout including TOT discrimination				
Crystal material and size	No. of channels	TLV3801 thr. / mV (single SPAD amplitude $\approx$ 10 mV)	CTR$_\textsf{osci}$ (FWHM) / ps	CTR$_\mathsf {TOFPET2+HF}$ (FWHM) / ps
TAC LYSO:Ce,Ca				
2$\times$2$\times$3 mm$^{3}$	1	20	70 ± 1	n.a.
2$\times$2$\times$3 mm$^{3}$	1	40	70 ± 1	118 ± 6
2$\times$2$\times$3 mm$^{3}$	1	90	78 ± 1	121 ± 6
2$\times$2$\times$3 mm$^{3}$	1	200	90 ± 1	126 ± 6
3$\times$3$\times$19 mm$^{3}$	1	40	n.a.	161 ± 3
3$\times$3$\times$19 mm$^{3}$	1	90	n.a.	165 ± 3
3$\times$3$\times$19 mm$^{3}$	1	200	n.a.	182 ± 2
2$\times$2$\times$3 mm$^{3}$	1 of 16^‡^	40^*^	79 ± 3	175 ± 5
2$\times$2$\times$3 mm$^{3}$	1 of 16^‡^	40^†^	n.a.	131 ± 2
2$\times$2$\times$3 mm$^{3}$	1 of 16^‡^	90^†^	n.a.	134 ± 2
3.88$\times$3.88$\times$19 mm$^{3}$	1 of 16^‡^	40^*^	137 ± 2	n.a.
3.88$\times$3.88$\times$19 mm$^{3}$	16	40^*^	173 ± 4^x^	242 ± 27^xx^
3.88$\times$3.88$\times$19 mm$^{3}$	16	40^†^	n.a.	223 ± 17^xx^
3.88$\times$3.88$\times$19 mm$^{3}$	16	90^†^	n.a.	235 ± 16^xx^
3.88$\times$3.88$\times$19 mm$^{3}$	16	90	n.a.	240 ± 14^xx^
EPIC BGO				
2$\times$2$\times$3 mm$^{3}$	1	50		542 447 (33 %) / 939 (67 %)
2$\times$2$\times$3 mm$^{3}$	1 of 16^‡^	40^*^	359	n.a.

The errors given estimate the statistical error on results from single-channel measurements following a $\chi ^2$-distribution. The value and error stated for multi-channel configurations report the mean and standard deviation of the mean, computed from the CTR of the individual channels

${}^{\circ }$ This value improves to 109 ps (94 ps (56 %) / 266 (44 %)) if applying a timewalk correction

${}^{*}$ Evaluated on the timing channels only, i.e., the energy spectrum contained the TOT of the TLV3801 pulse (TOFPET2 ASIC operated in QDC mode)

^‡^ All 16 SiPMs bias, but only one of them coupled to a scintillator

^†^ Adapted board version with a low pass implemented in the signal paths of the energy channels (*C*=100 nF, *R*=100 $\Omega$) and a decoupling capacitor (*C*=270 pF) in the timing branch replacing the PZ filter

$^\text {x}$ Value evaluated on one channel only

$^\text {xx}$ Value and error stated report the mean and standard deviation computed from the CTR of the individual channels

### Investigation on the performance of single channels in 16-channel HF electronics

To investigate the functionality of the 16-channel board, additional measurements have been performed using the 4$\times$4 NUV-MT SiPM array, biasing all 16 SiPMs, but only coupling one 2$\times$2$\times$3 mm$^{3}$ LYSO:Ce,Ca crystal to one of the SiPMs, ruling out a deterioration solely due to electronic crosstalk between channels or the implementation of the 16-channel HF circuit. For a direct connection to the TOFPET2 ASIC, these measurements resulted in the same CTR as actual single-channel coincidence experiments. For an HF connection to the TOFPET2 ASIC, the performance was deteriorated by approximately 10 ps to 131 ps (FWHM) compared to the single-channel version with a CTR of to 121 ps (FWHM) (c.f. Table 1). Only operating the timing channels, the CTR decreased to 175 ps, which is again based on filtering on a raw energy value spectrum that corresponds to the TOT of the TLV3801 signal. A similar investigation was performed using oscilloscope readout for one channel of the multi-channel HF board. Here, the CTR was 79 ps for a 2$\times$2$\times$3 mm$^{3}$ LYSO:Ce,Ca crystal and 137 ps for a 3.88$\times$3.88$\times$19 mm$^{3}$ LYSO:Ce,Ca crystal coupled to one of the SiPMs, respectively (c.f. Table 1). The deterioration between the values is similar to the deterioration in comparable configurations with TOFPET2 readout. Additionally, the CTR of a single channel of the 16-channel, one-to-one coupled detector block was measured with HF readout and digitization via the oscilloscope and is 173 ps (FHWM) as reported in Table 1.

### 16-channel HF electronics with a one-to-one coupled LYSO:Ce,Ca detector block

Moving to a 4$\times$4 matrix of one-to-one coupled, isolated 3.8$\times$3.8$\times$19 mm$^{3}$ LYSO:Ce,Ca crystals, an average CTR of 252±15 ps (FWHM) (default configuration, R$_\textsf{in}$ = 11 $\Omega$, c.f. Fig. 8a) and 219±24 ps (FWHM) (optimized configuration, c.f. Fig. 8b) was measured using a DC connection to the TOFPET2 ASIC. This is a deterioration of 60 ps to 80 ps compared to prior single-channel experiments, where a CTR of about 160 ps (FWHM) was measured (c.f. Table 1). Incorporating the 16-channel HF electronics including TOT discrimination in the signal path, an average CTR of 240±14 ps (FWHM) was achieved configuring a TLV threshold of 90 mV and 242±27 ps (FWHM) with a TLV threshold of 40 mV (c.f. Fig. 8c), which is, within its errors, in accordance with the aforementioned performance of the DC readout. Reducing the gain $\mathsf {G_T}$ of the transimpedance amplifier in the timing branch to diminish effects of noise on the timing trigger did not improve the CTR any further, as also seen in measurements exposing the SiPMs to laser pulses. The latter performance at a TLV threshold of 40 mV was evaluated only on the timing channels of the circuit, meaning the digitized raw energy values correspond to the TOT of the TLV3801 signal. Disconnecting the energy channels (originally connected of the ASIC front-end board (FEB/A) to the SiPM anodes) significantly reduced the instabilities and oscillations observed, which were even stronger than in single-channel experiments, and enabled this measurement at a TLV threshold of 40 mV. This hints at a feedback loop between the FEB/A and the 16-channel HF readout, which can be improved by capacitive decoupling. Consequently, adapting the filter parameters in the signal paths of the timing and energy channels improved the achievable CTR to 223±17 ps with a TLV threshold of 40 mV due to an improved energy resolution, which is the same as for a direct connection of the TOFPET2 input. Again, a higher threshold deteriorated the CTR, as seen for single-channel experiments, to 235±16 ps with a TLV threshold of 90 mV (c.f. Table 1 and Fig. 13).

**Fig. 9:**
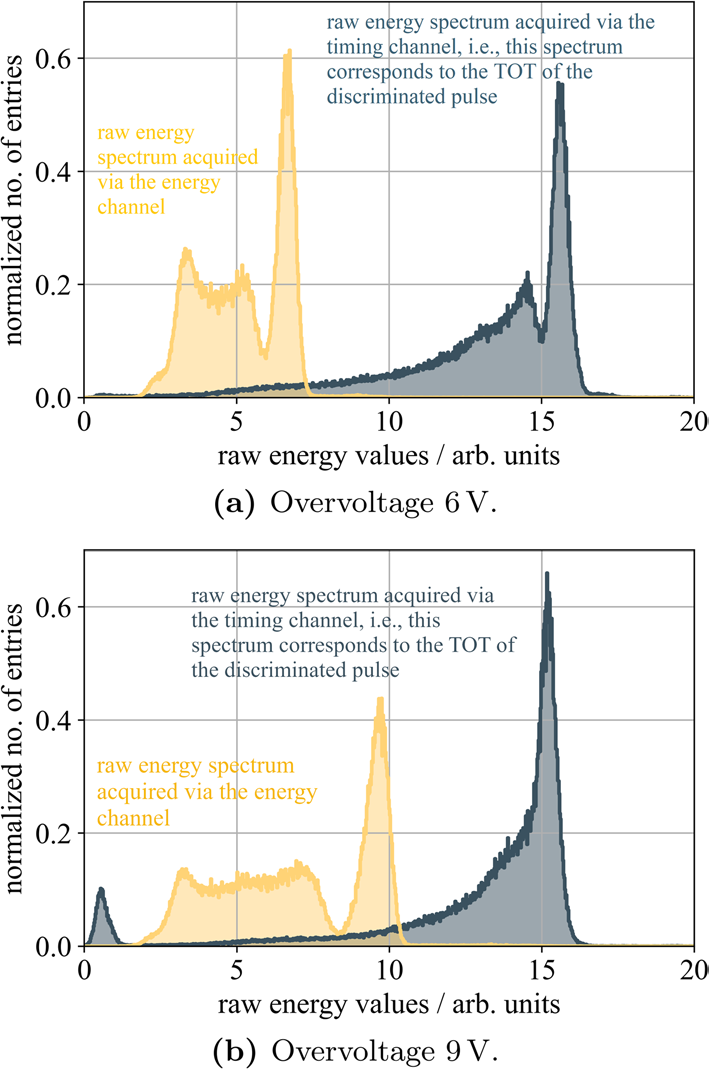
Channel raw energy value spectrum for a 2$\times$2 $\times$3 mm$^{3}$ LYSO:Ce,Ca crystal read out via one Broadcom NUV-MT SiPM channel of a 4 $\times$ 4 SiPM array connected to the 16-channel HF readout board at an overvoltage of 6 V and 9 V. Raw energy values were digitized via the TOFPET2 ASIC operated in qdc mode

**Fig. 10:**
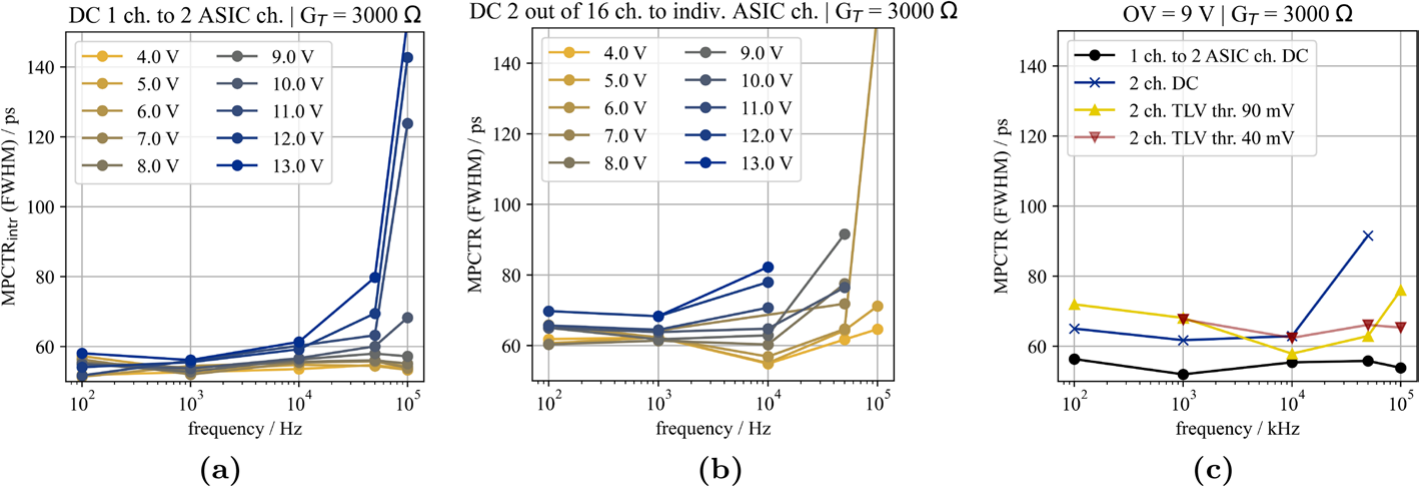
Multi-photon coincidence time resolution of the different SiPM-ASIC channel combinations illuminated with optical photons at 406 nm and at different frequencies. Data were acquired using Broadcom NUV-MT SiPMs and the TOFPET2 ASIC with an ASIC threshold of $\mathsf {vth\_t1} = 20$. **a** One SiPM channel coupled to two ASIC channels of the same ASIC. **b** Two SiPM channels on one SiPM array each coupled to one ASIC channel of the same ASIC. **c** Comparison between different combinations and readout electronics at an overvoltage of 9 V

**Fig. 11:**
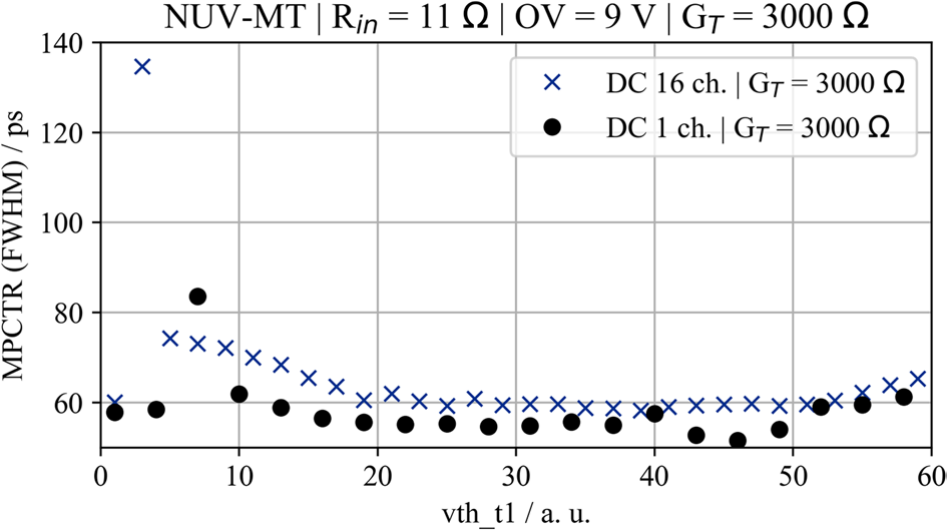
Coincidence multi-photon time resolution of the different SiPM-ASIC channel combinations illuminated with optical photons at 406 nm and a pulse frequency of 10 kHz and triggering at different TOFPET2 ASIC thresholds $\mathsf {vth\_t1}$. Data were acquired using Broadcom NUV-MT SiPMs and a DC connection to the TOFPET2 ASIC

### Energy resolution with and without linearized TOT

Currently, the multi-channel HF readout approach suggests the digitization of the signal’s energy via a separate channel, leading to an increase in the number of required readout channels, which could be considered problematic on system level in terms of form factor and power consumption. Two example energy spectra are shown in Fig. 9. Digitizing the energy via a TOT of the discriminated signal might deteriorate the energy resolution, which for a separate energy channel should remain as high as reported in [25]. A linearized TOT has therefore been tested in this initial design, which allows to improve the linearity ratio of the energy spectrum from 0.97 to 0.89. The energy resolution obtained in linearized TOT mode is 15.7 %, convolved with the TOFPET2 TDC resolution, compared to 10.1 % in TOFPET2 charge integration mode, both measured at an overvoltage of 9 V. Even without linearization, the quality of the raw energy value spectra acquired using only the timing channels allows to reliably separate Compton-scattered events from true coincidences and maintains the selection of events $\pm 2\sigma$ around the photopeak. As Fig. 9 shows that the peak separability decreases for higher overvoltages, each detector configuration requires at least a bias scan to evaluate the optimum point of performance with respect to peak separability and achievable timing performance.

### Timing limits with optical illumination using a pulsed picosecond laser

With a Broadcom AFBR-S4N33C013 SiPM coupled to an LYSO crystal (EPIC Crystal; 2$\times$2$\times$3 mm$^{3}$) connected to two channels of the same ASIC, as shown in Fig. 5a, the intrinsic CTR of the TOFPET2 ASIC was measured to be on average CTR$_\textsf{intr}$=58 ps (FWHM) at a threshold of $\mathsf {vth\_t1} = 20$ over the whole bias range scanned [25]. Illuminating an NUV-MT SiPM (c.f. Fig. 5b) with the laser, a value of MPCTR$_\textsf{intr}$=55 ps (FWHM) could be confirmed for several pulse frequencies in a range of 0.1 kHz to 100 kHz and bias voltages applied to the SiPM from 36.5 V to 45.5 V (corresponding to overvoltage 4 V to 13 V; c.f. Figure 10a). Connecting each of two SiPM channels to one ASIC channel on the same ASIC, as shown in Fig. 5c, slightly elevated the MPCTR value to about 65 ps to 70 ps (FWHM) measured with the standard readout with the TOFPET2 ASIC (c.f. Fig. 10b). The CTR shows a strong dependency on the overvoltage over the whole range of investigated frequencies. Reaching an MPCTR close to the intrinsic performance limit CTR$_\textsf{intr}$ at 4 V suggests a severe problem with baseline shifts at the TOFPET2 input stage limiting the ASIC’s performance at optimum SiPM operation point, i.e., a higher overvoltage. This results in a contribution of up to more than 80 ps (FWHM) to the overall CTR, depending on the chosen overvoltage. Additionally, the contribution of the front end was measured for the multi-channel HF readout board using TLV thresholds of 40 mV and 90 mV (c.f. Fig. 10c), which shows the same limitation as for the DC TOFPET2 readout at a fixed overvoltage of 9 V. A comparison of the achieved MPCTR$_\textsf{intr}$ and MPCTR over a range of TOFPET2 trigger thresholds is provided in Fig. 11. Here, it becomes evident that configuring sufficiently high trigger threshold (starting from $\mathsf {vth\_t1} = 20$) allows to converge to a MPCTR closer to the intrinsic timing limits of the TOFPET2 ASIC and reduce the contribution of the electronic front end to the overall MPCTR.

Switching to the 16-channel HF readout electronics including TOT discrimination prior to the TOFPET2 front end pushes the MPCTR of the 4$\times$4 NUV-MT SiPM array illuminated with a laser toward the intrinsic timing limit and results in about the same contribution of the front end of 70 ps (FWHM) over the entire frequency range, but reduces the deterioration at higher frequencies and higher overvoltages (c.f. Fig. 10c).

**Fig. 12:**
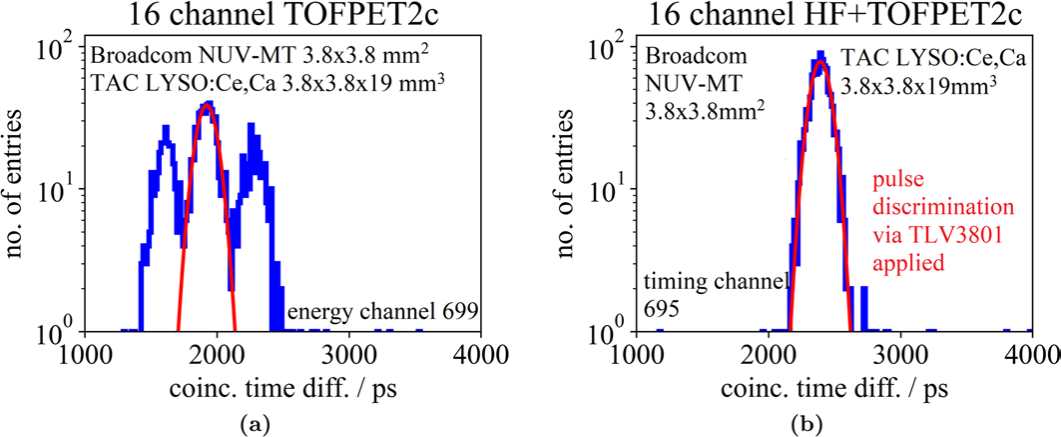
Channel coincidence time difference spectrum for the detector block read out via the TOFPET2 ASIC (left) and for the detector block read out via the 16-channel HF readout board including TOT discrimination with the TOFPET2 ASIC emulating the TDC (right) at an overvoltage of 9 V

**Fig. 13:**
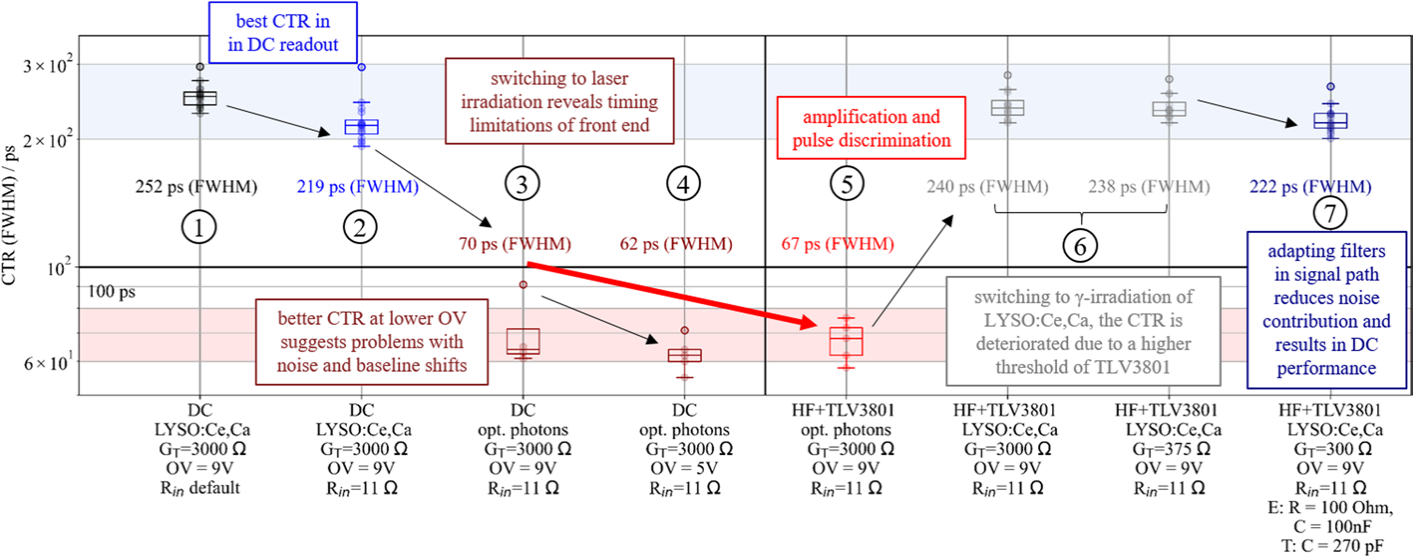
TOFPET2 performance of a detector block consisting of a $4\times 4$ Broadcom NUV-MT SiPM arrays one-to-one coupled to a $4\times 4$ LYSO:Ce,Ca crystal matrix in comparison with exposing the SiPMs to laser pulses (406 nm) of an SiPM array. The performance with default configuration and DC readout can be optimized (1), (2), while laser pulses reveal timing limitations of the front end (3). Reducing the applied bias leads to a better intrinsic resolution (4), which is why baseline shifts are thought to be the cause of the problem. Pulse amplification and discrimination via a TLV3801, i.e., 16-channel HF electronics, are employed (5), delivering the same intrinsic timing resolution at higher overvoltages. Switching back to a full detector block with a scintillator, the performance is still limited by a high TLV threshold, which is needed due to unstable baselines at lower thresholds. (6). Adapting signal filters in the timing and energy signal paths reduces this noise and improve the CTR to DC level (7), which shows that the adapted HF readout board does not worsen the CTR and, hence, could be a good candidate for readout with the picoTDC

## Discussion

### Impact of the TLV and the scaling of the circuitry on the CTR

Including the TLV3801 into the HF readout circuit only resulted in a marginal deterioration of the CTR from 58 ps (FWHM) to 70 ps (FWHM). The slight deterioration compared to previous studies can be attributed due to using only one amplifier stage and therefore triggering on a relatively higher leading-edge threshold. It is concluded that the additional signal processing, i.e., the TLV, has a small to negligible impact on the performance of crystal and SiPM.

Multi-channel measurements may suffer from additional deterioration due to light-sharing and crosstalk, where the latter is enhanced by mounting an optical coupling material and/or scintillator on top. This was investigated and confirmed by coupling a single small-sized crystal (TAC LYSO:Ce,Ca; 2$\times$2$\times$3 mm$^{3}$) to one of the 16 NUV-MT SiPMs.

### Effects impacting the multi-channel performance of the 16-channel HF readout

If an entire detector block (i.e., a matrix of 16 crystals) was coupled to the SiPM array, but only one channel was read out via the oscilloscope, the CTR deteriorated to 173 ps (FWHM) for one of the 3.88$\times$3.88$\times$19 mm$^{3}$ LYSO:Ce,Ca crystals. Again, the reason for this additional deterioration might be an increased amount of light-sharing and crosstalk due to optical coupling material and scintillator on top of multiple channels. These values only have been evaluated using the TOT information for event selection; hence, a worse peak separability in one of the cases can be excluded as the cause of the deterioration. Hence, the large deterioration of the CTR in multi-channel experiments with the TOFPET2 ASIC to about 220 ps (FWHM) is attributed to the aforementioned light-sharing and crosstalk effects and might as well stem from electronic crosstalk, unstable baselines caused by the aforementioned phenomenons or other effects of operating multiple channels on the same ASIC at once. Still, the obtained values are an improvement compared to prior studies using the TOFPET2 ASIC with SiPM arrays from different vendors [22].

The general idea of using the 16-channel circuit with BGO crystals is to demonstrate benefit from the detection of prompt Cherenkov photons in multi-channel applications. However, due to baseline shifts and an insufficient energy resolution based on the TOT at very low thresholds, the circuit currently does not allow to trigger on sufficiently low thresholds to enable such a measurement. These measurements will become the focus of interest once the combination with the picoTDC has been push forward.

### Time resolution limits of the TOFPET2 ASIC

Experiments with optical photon pulses showed an increased contribution of the TOFPET2 front-end electronics to the overall CTR, if the SiPMs were biased with higher voltage and if a higher pulse frequency was applied. This can be attributed to a higher probability for dark counts, crosstalk and therefore baseline shifts, which deteriorate the intrinsic performance of the TOFPET2 ASIC. Implementing TOT discrimination prior to the TOFPET2 input stage, as done for the 16-channel version of the HF readout electronics, filters these triggers from the ASIC input and, by routing rectangular and short LVDS signals, result in a more stable channel baseline without undershoots (c.f. Fig. 3). In single-channel coincidence experiments with $\gamma$-radiation, the 16-channel HF readout circuit achieved comparable results to prior single-channel HF readout versions, reaching CTRs in the order of 60 ps to 70 ps (FWHM). Adding the measured intrinsic resolution of the TOFPET2 ASIC, i.e., 70 ps (FWHM), and the CTR limits of crystal and SiPMs in square [25], the measured CTR of 118 ps (FWHM) almost matches the expected CTR of 99 ps (FWHM), showing that the TOT discrimination is reducing the impact of noise and baseline shifts on the TOFPET2 input stage and therefore on the ASICs performance at higher overvoltages. This is confirmed by a reduction of the side peaks in the coincidence time difference spectrum (c.f. Fig. 12), which were initially characterized in [25].

### Scalability of the design

We have shown a 16-channel implementation of emerging HF readout electronics. While the functionality of the design has been successfully tested, the scalability is currently limited by the high form factor of the discrete components used. Apart from reducing the power consumption [15], sophisticated shielding techniques may come into play to reduce the interference between components. First shielding attempts have been demonstrated using caskets [15, 16, 46]. Final 3D-integration could be facilitated by omitting the balun transformer as proposed in [15].

### Summary of points discussed

Figure 13 summarizes the results obtained in this study, showing a performance improvement from default to optimal readout with a direct connection to the TOFPET2 ASIC (c.f. Fig. 13 (1,2)). Exposing the bare SiPMs to laser pulses, the timing limitation due to the front end (with a slight influence from the employed SiPMs) was measured to 70 ps (FWHM) for DC readout (c.f. Fig. 13 (3)), which can be reduced 62 ps (FWHM) by a lower bias voltage applied to the SiPM array (c.f. Fig. 13 (4)), supporting the hypothesis of a strong impact of noise and baseline shifts. With TOT discrimination via a TLV3801, the SiPMs can be operated at higher bias voltage again, maintaining the timing resolution of 67 ps (c.f. Fig. 13 (5)). Regarding a slight deterioration of the performance with the 16-channel HF electronics compared to optimized DC readout (c.f. Fig. 13 (6)), one has to keep in mind the considerably high trigger threshold of the TLV3801, which was shown to deteriorate the TOF resolution in single-channel experiments mainly caused by photon statistics [47]. To mitigate shifts of the TOFPET2 channel baselines, the threshold has to be kept this high in multi-channel experiments. At lower TLV thresholds, oscillations could be reduced by disconnecting the energy channels. This shows that the ASIC front-end board might cause feedback signals, which result in instabilities and performance deterioration. Adapting filters in the signal paths of the timing and energy channel reduced the charge contribution of dark counts and resulted in a CTR that is equal to the one achieved for a direct connection (c.f. Fig. 13 (7)).

## Conclusion and outlook

A 16-channel HF readout concept including TOT discrimination has been implemented and tested successfully, achieving state-of-the-art CTRs and being able to mitigate the effects of noise and baseline shifts on the TOFPET2 front end, here used as initial test back-end electronics. The CTR measured in single- and multi-channel experiments is on par with the timing performance achieved with a direct connection to the TOFPET2 input stage, which suggests that the readout approach does not affect the timing performance of the back-end electronics. Still, the limitation the TOFPET2 front end is imposing on the achievable TOF resolution, despite a high single-channel intrinsic TOF resolution of 58 ps, and could not be overcome by this readout approach, as the circuit suffers from unstabilities at low TLV thresholds, due to the additional direct connection of the SiPM matrix with the TOFPET2 ASIC. We have found that direct connections to the front-end board could cause feedback signals which are the cause for these instabilities. Only using the HF amplification and TOT discrimination solves this problem, concluding that the multi-channel HF readout proposed in this study is stable in operation. To further push the achievable CTR, the design of an improved board version as well as the integration with novel digitization electronics with unprecedented timing precision, such as the picoTDC developed at CERN [34], is in order. This circuit will improve the intrinsic timing resolution of the electronic front end by at least a factor of 5 compared to the TOFPET2 ASIC and will therefore overcome one of the limitations of the current multi-channel readout. It is expected to realize TOF resolutions close to the limits of photosensor and scintillator measured with the oscilloscope. A combination of this 16-channel HF readout with previous studies on TOF- and depth-of-interaction-capable detector units is as well planned [46]. Overall, it can be concluded that the presented electronic front end does not affect the timing performance of the back-end electronics used in single- and multi-channel experiments and is hence a promising readout approach to be connected to ultrafast, precise back-end electronics.

## Data Availability

The datasets used and/or analyzed during the current study are available from the corresponding author on reasonable request.
